# Automation of Workplace Lifting Hazard Assessment for Musculoskeletal Injury Prevention

**DOI:** 10.1186/2052-4374-26-15

**Published:** 2014-06-24

**Authors:** June T Spector, Max Lieblich, Stephen Bao, Kevin McQuade, Margaret Hughes

**Affiliations:** 1 Department of Environmental & Occupational Health Sciences, University of Washington, 4225 Roosevelt Way NE, Suite 100, Seattle, WA 98105, USA; 2 Department of Medicine, University of Washington, 4225 Roosevelt Way NE, Suite 100, Seattle, WA 98105, USA; 3 Department of Mathematics, University of Washington, Seattle, WA, USA; 4 Safety and Health Assessment and Research for Prevention (SHARP) Program, Washington State Department of Labor and Industries, Olympia, WA, USA; 5 Department of Rehabilitation Medicine, University of Washington, Seattle, WA, USA

**Keywords:** Back injury, Back pain, Depth camera, Ergonomics, Microsoft Kinect, Musculoskeletal hazard assessment, NIOSH lifting equation, Prevention, Work-related musculoskeletal disorders

## Abstract

**Objectives:**

Existing methods for practically evaluating musculoskeletal exposures such as posture and repetition in workplace settings have limitations. We aimed to automate the estimation of parameters in the revised United States National Institute for Occupational Safety and Health (NIOSH) lifting equation, a standard manual observational tool used to evaluate back injury risk related to lifting in workplace settings, using depth camera (Microsoft Kinect) and skeleton algorithm technology.

**Methods:**

A large dataset (approximately 22,000 frames, derived from six subjects) of simultaneous lifting and other motions recorded in a laboratory setting using the Kinect (Microsoft Corporation, Redmond, Washington, United States) and a standard optical motion capture system (Qualysis, Qualysis Motion Capture Systems, Qualysis AB, Sweden) was assembled. Error-correction regression models were developed to improve the accuracy of NIOSH lifting equation parameters estimated from the Kinect skeleton. Kinect-Qualysis errors were modelled using gradient boosted regression trees with a Huber loss function. Models were trained on data from all but one subject and tested on the excluded subject. Finally, models were tested on three lifting trials performed by subjects not involved in the generation of the model-building dataset.

**Results:**

Error-correction appears to produce estimates for NIOSH lifting equation parameters that are more accurate than those derived from the Microsoft Kinect algorithm alone. Our error-correction models substantially decreased the variance of parameter errors. In general, the Kinect underestimated parameters, and modelling reduced this bias, particularly for more biased estimates. Use of the raw Kinect skeleton model tended to result in falsely high safe recommended weight limits of loads, whereas error-corrected models gave more conservative, protective estimates.

**Conclusions:**

Our results suggest that it may be possible to produce reasonable estimates of posture and temporal elements of tasks such as task frequency in an automated fashion, although these findings should be confirmed in a larger study. Further work is needed to incorporate force assessments and address workplace feasibility challenges. We anticipate that this approach could ultimately be used to perform large-scale musculoskeletal exposure assessment not only for research but also to provide real-time feedback to workers and employers during work method improvement activities and employee training.

## Introduction

Work-related musculoskeletal disorders (WMSDs) are associated with substantial morbidity and cost. The economic burden of WMSDs in the United States (US) alone is estimated to be between $45 billion and $54 billion annually [1]. Evaluation of WMSD risk factors at the population level, with the ability to provide near real-time feedback to workers and employers, may facilitate effective WMSD prevention. However, existing methods for evaluating musculoskeletal exposures, including posture, repetition, and force, in workplace settings have limitations [2]. These limitations include high cost and complexity (e.g. three dimensional [3D] motion capture systems with markers), interference by large metal objects in the industrial environment (e.g. magnetic tracking and inertial measurement systems), invasiveness or limited practicality in workplace settings (e.g. electromyography, electro-goniometers, force plates), and resource intensiveness when deployed in large working populations (e.g. observational analysis of recorded two dimensional [2D] video data) [3-11] .

Recent advances in machine learning (ML) and computer vision offer the promise of being able to collect and report musculoskeletal exposure and task information in a semi-automated, non-invasive (*without* markers or sensors applied to the body), and near real-time fashion, and to predict WMSDs from musculoskeletal exposure data. There are a growing number of ML applications, including applications in workplace settings to predict non-WMSD injuries from information about safety hazards [12].

Computer vision is the subfield of computer science devoted to processing, recognizing, and responding to 2D or 3D image data, as collected by digital cameras or devices like the Microsoft Kinect [13] or ASUS Xtion [14]. The Kinect and Xtion, which were originally developed for video gaming and are relatively inexpensive (several hundred US dollars each), consist of a red-green-blue camera and 3D infrared depth sensor (RGB-D) that can collect position (e.g. Cartesian coordinate) and color data at approximately 30 frames/second. This hardware can be used with available software development kits and other open source tools to develop applications. Such applications are being increasingly studied in health research, including in areas of gait analysis [15] and neurological rehabilitation [16]. However, little work has been published to date on the use of depth camera systems in occupational health [2].

We aimed to automate the estimation of parameters used in the revised US National Institute for Occupational Safety and Health (NIOSH) lifting equation with the Microsoft Kinect. The lifting equation is a standard manual observational tool used to evaluate back injury risk related to manual lifting in workplace settings [9]. The equation is based upon biomechanical, physiological, and psychophysical criteria, and informed by an extensive review of the literature on low back pain [9]. The lifting equation is reasonably well-correlated with risk of certain types of musculoskeletal disorders [17], but it can be difficult to apply by those who have not received specific training [18], and some of the equation’s inputs involve time-consuming measurements. In addition, most workplace lifting tasks are complicated, for example with respect to different levels and reaches, and currently used observation methods require users to make assumptions that may not reflect the complexity of the lifts.

One possible approach for improving the practicality the lifting equation is to use the Microsoft Kinect skeleton algorithm to automatically estimate lifting equation parameters using estimated joint positions. The Kinect skeleton algorithm employs random forests, trained using large sample datasets, to segment the entire depth image into body parts [19]. For each body segment, it uses mean shift techniques and a learned joint center offset to estimate the joint center position. Although the Kinect skeleton algorithm is designed to work quickly and relatively robustly [19], the lack of an underlying kinematic model and the static, frame-by-frame nature of the algorithm result in some inaccuracies in posture estimation [20,21]. To address this limitation, we assembled a dataset of lifting tasks and other motions, simultaneously recorded in a laboratory setting by the Kinect and by a standard optical motion capture system. We used these data to develop error-correction regression models to improve the accuracy of NIOSH lifting equation parameters.

In this paper, we report our tool’s accuracy in estimating NIOSH lifting equation parameters, compared with a standard optical motion capture system. We also discuss the feasibility of the tool in workplace settings.

## Material and methods

### Subjects and setting

To develop error-correction regression models, we first collected human motion data in the Motion Analysis Laboratory in the University of Washington Medical Center, Department of Rehabilitation Medicine. Six subjects (four males, two females; mean age 25 years) were fitted with reflective markers and performed several trials in the laboratory, while being simultaneously recorded by the Kinect (Microsoft Corporation, Redmond, Washington, United States) and an eight-camera optical motion capture system (Qualysis, Qualysis Motion Capture Systems, Qualysis AB, Sweden). Each trial started with a synchronization gesture used to correlate the Kinect and Qualysis recordings. The trials consisted of walking, upper arm movement, “random” motion in the capture volume, and lifting an object from the floor to a waist-height table. We included tasks and motions other than lifting in the trials in order to sample from the largest possible space of positions and build more robust models. The University of Washington Institutional Review Board approved the study protocol, and each participant provided written informed consent prior to participation.

### Data processing

**Figure 1:**
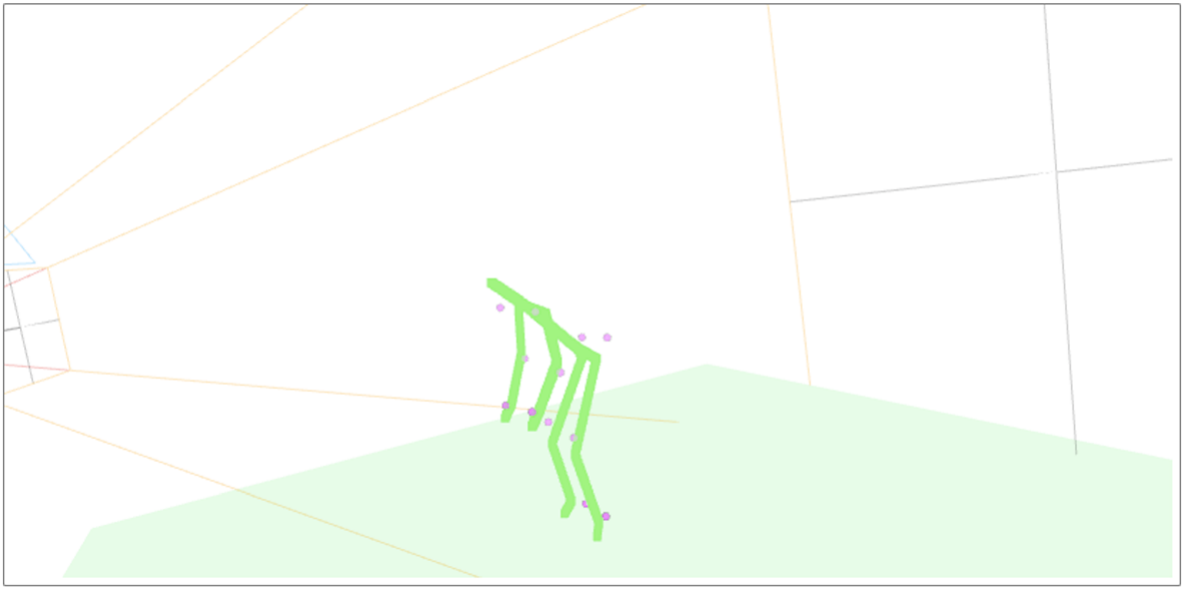
Web-based software tool visualization of Qualysis and Kinect skeleton algorithm output of a lifting trial (Dots = Qualysis estimates; Lines = Kinect estimates).

Kinect data were processed by a Windows Presentation Foundation application using the Microsoft Application Programming Interface (API), and skeleton coordinates were extracted. Qualysis coordinate data were exported as C3D files, and a musculoskeletal model was developed using MotionMonitor software (Innovative Sports Training, Inc., Chicago, Illinois, United States). Outputs of the MotionMonitor model included 3D coordinates of anatomical joint centers, as well as local quaternions. The data were filtered with a 4th order recursive low-pass Butterworth filter with a 7 Hz cutoff. Kinect data were then further smoothed with a Savitzsky-Golay filter of degree three with window of size 33 (approximately one second). Kinect and Qualysis data were then subsampled at uniform times, and cross-correlation of the right wrist Z-coordinate was used to align the recordings. A web-based software tool was developed using Python, Node.js, and WebGL to visually compare Kinect and Qualysis data (Figure 1), and obvious outliers were excluded from the data set by hand. A total of roughly 22,000 usable frames remained after this “hand-cleaning” of the data. Each frame consists of a pair (K, Q), where K is a set of coordinates for a skeleton as reported by the Kinect algorithm, and Q is the coordinates of the skeleton as produced by MotionMonitor software from the Qualysis input data.

### NIOSH lifting equation

Using metric units, the revised NIOSH lifting equation can be written: RWL = LC * HM * VM * DM * AM * FM * CM, where:

• RWL denotes the Recommended Weight Limit (the weight of the load, in kg, that nearly all healthy workers could perform over a substantial period of time without increasing the risk of developing low back pain)

• LC is the Load Constant (23 kg)

• HM is the Horizontal Multiplier, calculated as 25/H (evaluated at the beginning of the lift), where H is the distance, in cm, between the mid-point of the line joining the ankles to a point projected on the floor directly below the mid-point of the hands.

• VM is the Vertical Multiplier, calculated as 1 – (0.003 * |V – 75|) (evaluated at the beginning of the lift), where V is the vertical height of the hands above the floor, in cm.

• DM is the Distance Multiplier, calculated as 0.82 + 4.5/D, where D is the vertical travel distance of the hands between the origin and destination of the lift, in cm.

• AM is the Asymmetry Multiplier, calculated as 1 – 0.0032A, where A is the angle, in degrees, between the asymmetry line (the horizontal line that joins the mid-point between the ankles and the point projected on the floor directly below the mid-point of the hands) and the mid-sagittal line.

• FM is the Frequency Multiplier, drawn from an empirically determined table (derived from the frequency of lifts per minute), and

• CM is the Coupling Multiplier, drawn from an empirically determined table (derived from the pliability of the material making up the load being lifted, classified as good, fair, or poor) [9].

The Lifting Index (LI) = Load Weight/RWL provides a relative estimate of the physical stress associated with a manual lifting task. An LI greater than 1.0 indicates an increased risk for lifting-related low back pain [9].

### Model building

In building error-correction models, we focused on NIOSH lifting equation parameters H, V (evaluated at the origin and destination of the lift to compute D), and A. We aimed to develop models that would allow prediction of Qualysis values (taken to be the ground truth) from Kinect values. For each data point (K, Q), we modeled the following errors: ***δ***_*x*_*(K) = x(Q) - x(K),* where *x(Q)* and *x(K)* and denote the parameters *X* (e.g. H, V, A) calculated from Qualysis and Kinect joint positions, respectively. We imposed a coordinate system on the joint positions similar to that used in [22]:

• the origin is the average of the joint centers for both shoulders and hips;

• the Z-axis is the true vertical direction in the room at the time of measurement;

• the X -axis is the horizontal component of the vector normal to the plane spanned by the Kinect right and left hip and hip center joints;

• the Y-axis is chosen to make a right-handed coordinate system.

Data mirrored across the XZ-plane were included in the dataset in order to compensate for asymmetries in the data collection. Each ***δ***_*x*_ is assumed to be a noisy sample from a function defined on the space of Kinect positions with the above coordinate system. We used gradient boosted regression trees with a Huber loss function to generate a model for each ***δ***_*x*_.

### Analyses

We determined H, V, and A at each time-point for lifting trials for three subjects (subjects #1, #2, #3) using raw Kinect skeleton data (“raw”) and data modeled using our error correction model (“modeled”). For each parameter, we generated box plots of the error, as compared to parameters generated from Qualysis data. To avoid overly optimistic estimates for error correction of the modeled data, we estimated modeled parameters by training the models on data coming from all but one subject and testing the model on the excluded subject.

We also tested models on three lifting trials (trial #1, #2, #3) performed by two subjects not involved in generating the model building dataset. These subjects performed lifting tasks with specific *a priori* (“measured”) lifting equation parameters and frequencies. These lifting tasks were designed to be different (i.e. have different parameters) from the lifting motions used during data collection. Lifting cycle starts and stops were calculated using local maxima and minima for the mean Z-coordinate of the hands, filtered to retain only the frequency components corresponding to natural human lifting frequencies and then adjusted to align with local maxima and minima in the smoothed unfiltered data. The final values of parameters for these subjects were calculated as the mean over all of the recorded lifting cycles, using the inferred cycle starts and stops. For each subject, we report the raw, modeled, and measured parameters as well as the computed RWL.

All code was written in Python programming language, using numpy [23] and scikit-learn [24] for the development of the models and the parameter calculations.

## Results

### Model characteristics

**Figure 2:**
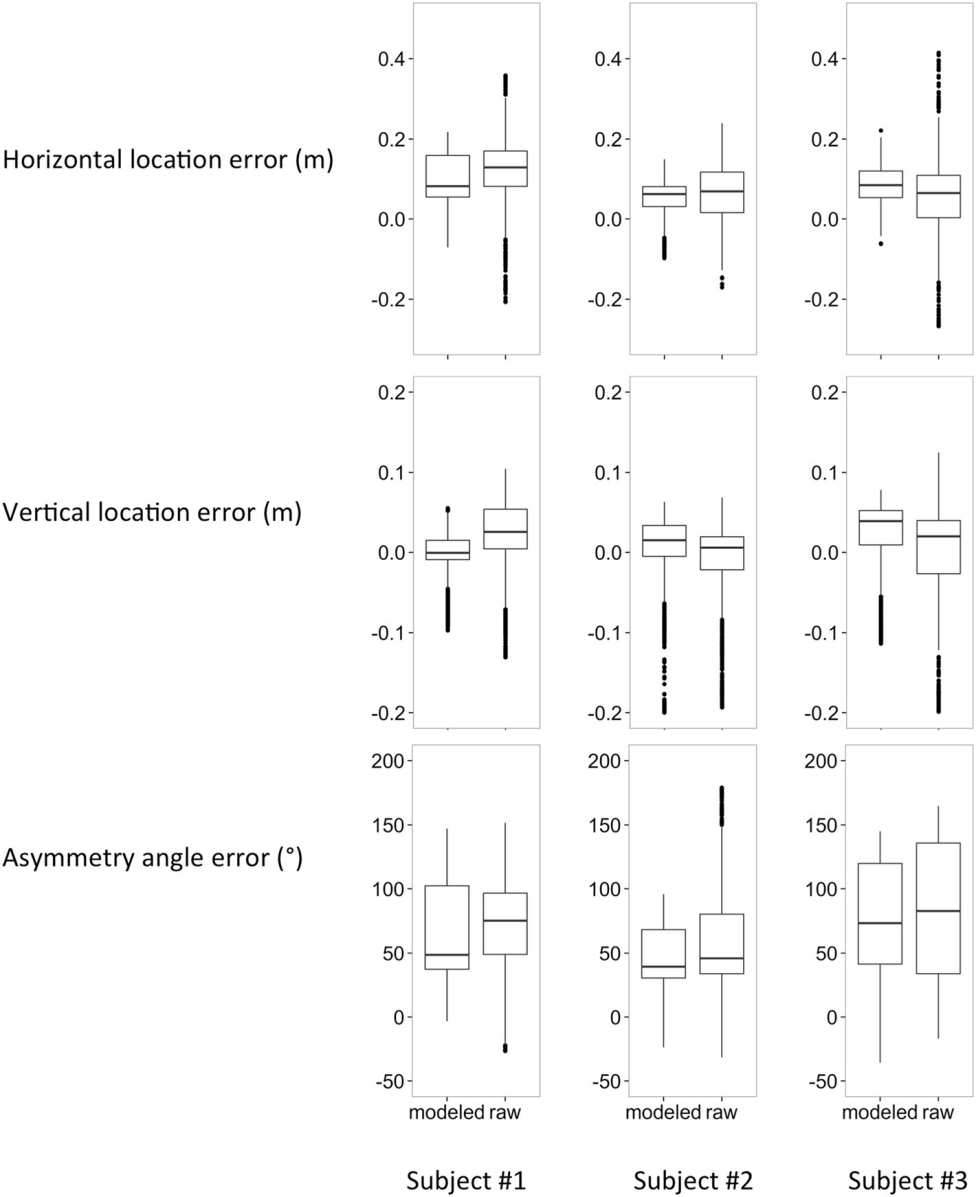
Box plots of errors for the horizontal location, vertical location, and asymmetry angle parameters for each subject.

**Figure 3:**
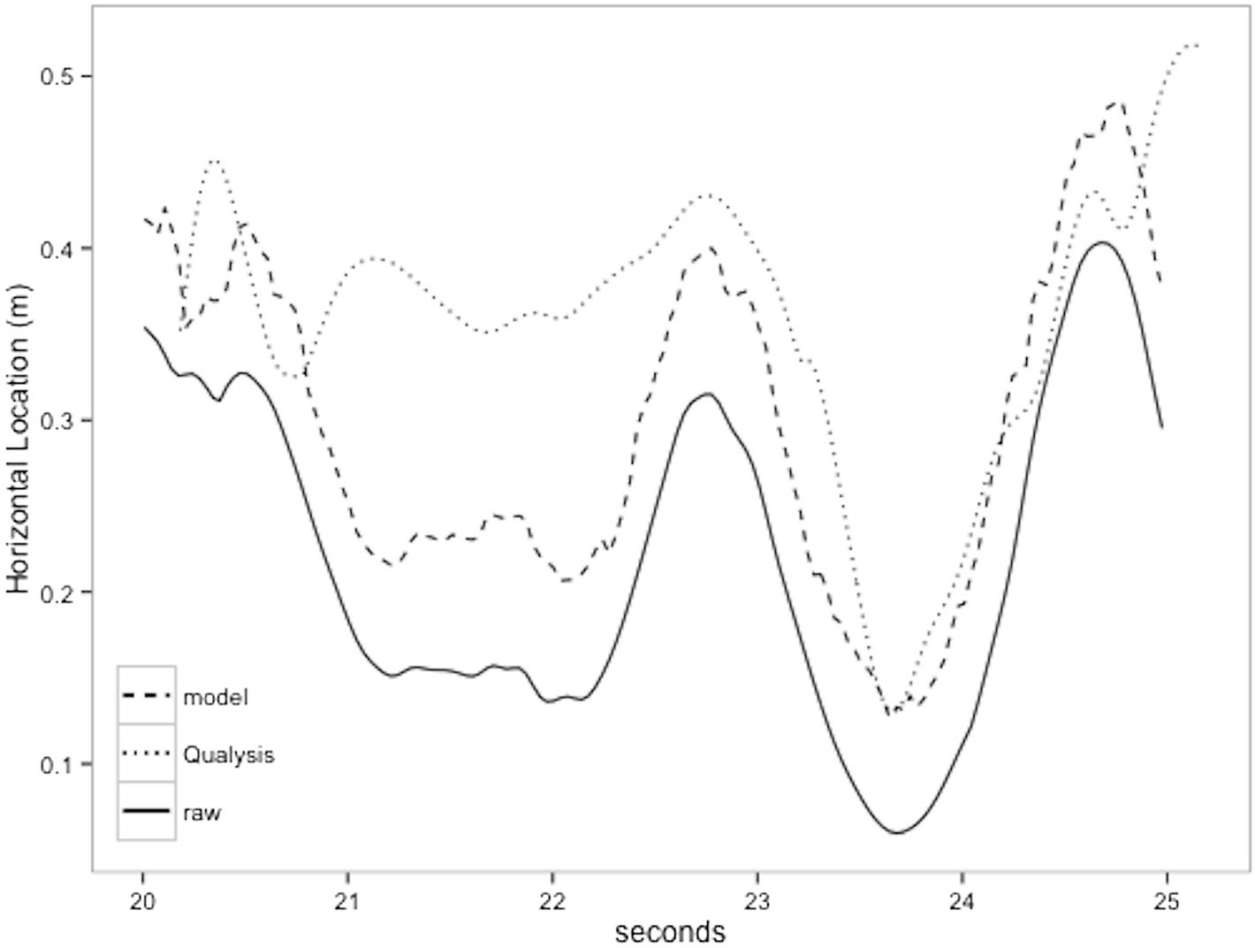
Horizontal location estimate (raw, modeled, Qualysis) over time for subject #1.

Box plots of errors for the horizontal location, vertical location, and asymmetry angle parameters for each subject are shown in Figure 2. In general, the variance of the errors for these parameters decreased after modeling. The Kinect underestimated parameters, and models generally reduced bias, as indicated by a smaller distance from zero, for the more biased parameter estimates. This phenomenon is particularly evident for subject #1’s horizontal location, vertical location, and asymmetry angle estimates.Figure 3 shows an example of subject #1’s horizontal location estimates (raw, modeled, Qualysis) over time. In general, the Kinect underestimated the horizontal location over time. The modeled estimates were, on average, closer to the Qualysis estimates.

### Model testing

Table 1 shows lifting equation parameters and RWLs estimated from testing our models on three lifting trials with *a priori* (“measured”) lifting equation parameters and frequencies, performed by subjects not involved in model building. RWLs derived from the measured data were 2.49 kg, 2.95 kg, and 2.95 kg for trials #1, #2, and #3, respectively. In general, use of raw Kinect skeleton data tended to overestimate the RWL for the three trials (4.90 kg, 3.07 kg, 3.57 kg, respectively), compared to the RWL derived from the measured data. The modeled data gave more conservative estimates that were lower than the RWLs derived from the measured data for the three trials (2.30 kg, 2.58 kg, 2.76 kg, respectively).

**Table 1:** Lifting equation parameters and recommended weight limits for external lifting trials

	**Trial #1**^**1**^			**Trial #2**^**1**^			**Trial #3**		
**Parameter**	**Measured**^**2**^	**Raw**	**Modeled**	**Measured**^**2**^	**Raw**	**Modeled**	**Measured**^**2**^	**Raw**	**Modeled**
H	0.48	0.29	0.50	0.61	0.56	0.67	0.61	0.54	0.66
V	0.07	0.00	−0.03	0.37	0.22	0.21	0.37	0.31	0.33
D	0.80	0.70	0.75	0.39	0.29	0.32	0.39	0.25	0.24
A	30	18	76	44	44	38	44	31	47
Frequency^2,3^	11	11	11	10	10	10	10	10	10
Coupling^2^	F	F	F	F	F	F	F	F	F
RWL	2.49	4.90	2.30	2.95	3.07	2.58	2.95	3.57	2.76

A = asymmetry angle (**°**); D = vertical distance (m); F = fair coupling; H = horizontal location (m); RWL = recommended weight limit (kg); V = vertical location (m).

^1^Same subject.

^2^Determined *a priori.*

^3^Cycles per minute.

## Discussion and conclusions

Our error-corrected Kinect-based automated lifting tool appears to produce estimates for the NIOSH lifting equation that are more accurate than those derived from the Microsoft Kinect algorithm alone. To date, there are few other reports of the use of an error-correction approach to optimize Kinect skeleton algorithm output [25], particularly for occupational health applications. Our results suggest that it may be possible to produce reasonable estimates of posture and temporal elements of tasks, such as task frequency, in an automated fashion.

Error correction of NIOSH lifting equation parameters estimated using the Kinect is particularly important for the horizontal location parameter, H. The inverse dependence of the RWL upon H makes the lifting equation calculation especially sensitive to this parameter. Consistent with previous studies, we observed that the parameter A is difficult to calculate both manually and by computer [26,27]. However, the parameter A has a lower impact on the RWL.

Use of raw Kinect skeleton data tended to overestimate the RWL, whereas our models gave more conservative estimates. This overestimation has important public health implications: lifting indices computed using raw Kinect data for loads between the measured and raw RWLs in our examples are less than or equal to 1.0, indicating no increased risk for lifting-related low back pain, whereas those calculated using our models are greater than 1.0, indicting an increased risk. In other words, use of raw Kinect skeleton data tended to result in falsely high recommended safe weight limits for loads, whereas our models gave more conservative, protective estimates.

### Limitations

Our models were built and cross-validated using 22,000 human pose samples drawn from laboratory time series (lifting motions, walking motions, arm motions, random motions). Because these recorded poses were not independent and identically distributed samples from the space of human postures, it is challenging to evaluate the true effectiveness of our modelling strategy using the statistics of our model performance on the collected data. Moreover, in this pilot study we only studied the validity of the models constructed from our 22,000 points on three external lifting task samples. The validity of our strategy needs to be confirmed in a larger study with a larger number of lifting samples and with randomized lifting parameters tested on a larger number of subjects doing similarly randomized lifting tasks. Our results suggest that a larger study is warranted.

Our study has several additional limitations: 1) we did not address force, a well-established factor associated with musculoskeletal disorders [1]; 2) we did not automate the estimation of several NIOSH lifting equation parameters, including the coupling multiplier; and 3) we developed and tested the models in a laboratory setting using simulated tasks, but workplace settings are substantially more complex. We suggest approaches to address these limitations in the subsequent subsection.

### Future directions

There are several possible approaches to incorporate force in our tool in an automated manner. First, additional computer vision techniques could be applied to our tool. For example, Eulerian Video Magnification, which tracks the variation of individual pixels over time and then exaggerates those differences, has been used to estimate heart rate from human skin color changes that occur with circulation [28]. Similar techniques could be used to characterize blanching and blushing responses that occur in the skin in response to mechanical loads [29]. However, this approach may be limited by personal protection equipment (e.g. glove) use and sensor resolution. Second, visual information about postural changes resulting from effort and exertion may be used to grossly classify loads into different categories, using machine-learning methods, from which force information could be inferred. These changes may only manifest after fatigue develops, however, and further work must be done to develop and assess the feasibility of this approach. Third, our tool could be combined with other sensors, such as surface electromyography or accelerometers, although this would necessitate instrumentation of the worker.

We did not automate the estimation of the coupling multiplier. The coupling component captures the nature of the hand-to-object gripping method: good coupling (e.g. lifting a box with hand-hold cut-outs) reduces maximum grasp forces required, while poor coupling (e.g. lifting a non-rigid bag that sags in the middle) generally requires higher maximum grasp forces [9]. It may be possible to use computer vision object recognition techniques [30] to automate the estimation of this parameter, or the user could manually enter this parameter value into our tool.

Even in workplaces that possess characteristics that are most compatible with depth camera use, such as the relatively common occurrence of stereotypical tasks in fixed locations, the Kinect algorithm may be limited. In a post-hoc analysis, we evaluated the feasibility of our tool in manufacturing workplace settings and found a high degree of occlusion (e.g. by other objects or workers and equipment) and postures that the Kinect algorithm was not designed to recognize (e.g. back to the camera). These factors significantly degrade Kinect-based posture estimates. Although the Kinect algorithm does attempt to infer the positions of occluded body parts, these inferences are rarely accurate. Further, workers moving too close or too far from the camera affected the accuracy of the skeleton algorithm. Our models did not perform optimally in these settings. To further optimize this tool, better worker capture algorithms need to be developed for potential use in conjunction with the methods presented here. We are currently developing alternative algorithms for detecting workers in complex workplace scenes and recognizing the posture of these workers using depth camera data. Improvements in depth camera hardware, such as in the Kinect for Windows V2 [13], may also be helpful.

Combining force and exertion data with posture and other temporal task data may allow the application of our methods to other musculoskeletal hazard assessment tools such as the American Conference of Governmental Industrial Hygienists Hand Activity Level Threshold Limit Value [7], the upper extremity Strain Index [8], and the Rapid Upper Limb Assessment [10]. Given the computational efficiency of our approach, we anticipate that our tool could ultimately be used to perform large-scale musculoskeletal exposure assessment not only for research but also to provide real-time feedback to workers and employers during work method improvement activities and employee training. Interventions that use the tool (e.g. for real-time feedback) could be tested in future studies for their effectiveness in preventing WMSDs.

## Abbreviations

(API): Application programming interface; (A): Asymmetry angle; (AM): Asymmetry multiplier; (CM): Coupling multiplier; (DM): Distance multiplier; (F): Fair coupling; (FM): Frequency multiplier; (H): Horizontal location; (HM): Horizontal multiplier; (LI): Lifting index; (LC): Load constant; (ML): Machine learning; (NIOSH): National Institute for Occupational Safety and Health; (RWL): Recommended weight limit; (RGB-D): Red-green-blue-depth; (3D): Three dimensional; (2D): Two dimensional; (US): United States; (D): Vertical distance; (V): Vertical location; (VM): Vertical multiplier; (WMSDs): Work-related musculoskeletal disorders.

## Competing interests

The authors declare that they have no competing interests.

## Authors’ contributions

JTS helped design and carry out the laboratory studies and drafted the manuscript; ML helped design and carry out the laboratory studies, conducted the analyses, and helped draft the manuscript; SB designed and helped carry out the laboratory studies and contributed to the manuscript; KM designed and carried out the laboratory studies and contributed to the manuscript; MH helped carry out the laboratory studies and contributed to the manuscript. All authors read and approved the final manuscript.
